# Optimizing Measurement of Vascular Endothelial Growth Factor in Small Blood Samples of Premature Infants

**DOI:** 10.1038/s41598-019-43108-7

**Published:** 2019-05-01

**Authors:** Claudia C. Lopez Yomayuza, Klaus T. Preissner, Birgit Lorenz, Knut Stieger

**Affiliations:** 1 Department of Ophthalmology, Justus-Liebig-University, 35392 Giessen, Germany; 2 Department of Biochemistry, Medical School, Justus-Liebig-University, 35392 Giessen, Germany

**Keywords:** Biomarkers, Paediatric research

## Abstract

To establish a method that allows for the reliable assessment of vascular endothelial growth factor (VEGF-A) concentrations in very small blood samples of preterm infants. Systemic VEGF measurements are important in view of the most appropriate Anti-VEGF drug to be used for the treatment of acute retinopathy of prematurity (ROP). Cord blood samples from preterm (n = 6) infants, blood samples from preterm infants with treatment requiring ROP (n = 12), and blood samples from healthy adults (n = 10) were collected. Serum, citrate plasma, and serum from recalcified citrate blood were obtained. Levels of VEGF-A and platelet factor-4 (PF-4) were quantified by ELISA or AlphaLISA immunoassay. VEGF-A levels could be detected by both assays, with the AlphaLISA generating slightly lower levels in healthy adults, but not in cord blood of preterm infants. In plasma samples, VEGF levels ranged from non detectable to 181 pg/ml. PF-4 concentrations were between 0.16–3.88 µg/ml. Values of VEGF-A and PF-4 in serum and recalcified serum were significantly higher compared to plasma through the release of these cytokines after platelet activation. In plasma samples of infants with ROP, VEGF-A could always be detected and its values ranged from 19.50 to 245.91 pg/ml and PF-4 concentrations were between 0.1 and 3.3 µg/ml. Using the AlphaLISA kit, we were able to detect VEGF in small sample volumes (5 µl plasma or serum/well) in premature infants with treatment requiring ROP and to monitor platelet activation by PF-4 detection. Minimal blood probe volumes reduce phlebotomy losses avoiding the risk of iatrogenic anemia, thus allowing close monitoring of the cytokine levels in these very fragile infants.

## Introduction

Retinopathy of prematurity (ROP) is a severe retinal neovascular disorder and an important cause of childhood blindness. During the pathologic process, the expression levels of several cytokines are affected, amongst which the vascular endothelial growth factor (VEGF) plays a crucial role. VEGF is a primordial growth factor for the normal vascularization process and its expression is regulated by hypoxia-inducible transcription factors^1,2^. While the VEGF family of proteins can be classified into at least seven members (VEGF-A, -B, -C, -D, -E, -F, and PlGF), it is VEGF-A that is clinically the most relevant protein with angiogenetic activity^3^. This glycoprotein is expressed in several isoforms with varying amino acid length (i.e. 111, 121, 145, 148, 162, 165, 183, 189, and 206 amino acids) generated from the same cDNA, and the affinity to the extracellular matrix inversely correlates with increasing length^4^. Major isoforms identified by mRNA expression analysis are VEGF121, 165, and 189^4^.

Since increased VEGF levels represent a major stimulus for neovascular insults to the retina, intravitreal application of anti-VEGF molecules such as Bevacizumab (Avastin, Genentech Inc), Ranibizumab (Lucentis, Novartis Pharma AG and Genentech Inc) or Aflibercept (Eylea, Bayer and Regeneron Pharmaceutical Inc) has been used to treat acute severe ROP^5–8^, after it had been demonstrated in a randomised trial that Bevacizumab was superior to conventional laser photocoagulation in zone I ROP^7^. However, particularly in premature infants suffering from severe ROP, the application of anti-VEGF molecules, in case of recurrences repeatedly, may pose a risk to the normal organogenesis. Recent data suggest a correlation of anti-VEGF administration in infants with ROP and a reduced neurodevelopment at 18 months corrected age^8^. Suppression of VEGF has been reported to depend on the specific anti-VEGF molecules used. On the other hand, faster clearance of Anti-VEGF molecules may be associated with higher needs for rescue and recurrence therapies. Therefore, precise knowledge about systemic VEGF levels is of high interest and crucial for performing and monitoring any treatment approaches.

Although of high interest, ocular fluid samples cannot be obtained as part of routine follow-up outside vitrectomies as this would put the infants unnecessarily at risk. Therefore, the concentrations of systemic VEGF and other angiogenic cytokines have been evaluated in a variety of different set-ups with the intention to identify either systemic predictors of pathogenic retinal angiogenesis or changes in expression of such factors as a consequence of systemic VEGF-blockage by intravitreal anti-VEGF therapy^9–14^. However, repeated phlebotomy loss in the first weeks of life is one of the major causes of iatrogenic anaemia, and can significantly contribute to the exposure to red blood cells transfusions^15,16^.

In plasma, VEGF-A levels represent the free circulating form of the cytokine, whereas in serum, an additional pool of platelet-derived VEGF-A contributes to the overall cytokine concentration. This is because VEGF-A, together with other growth factors such as platelet factor-4 (PF-4), angiopoietin-1 (ANG-1), insulin-like growth factor 1 (IGF-1), platelet-derived growth factor (PDGF), clotting proteins, or cytokines, is stored in platelet α-granules and becomes released upon activation^17,18^. Therefore, blood drawing procedures, which activate haemostasis and blood coagulation, lead to largely varying concentrations of VEGF-A in serum compared to plasma. In addition, different anticoagulants can also affect the measured VEGF-A levels in plasma. For example, the use of EDTA as an anticoagulant leads to considerable fluctuations of VEGF-A concentrations depending on the time between the drawing procedure and the measurement^17^. In contrast, due to the absence of platelet activation, concentrations measured in plasma prepared with sodium citrate do not change during the first two hours after the drawing procedure^17,19–21^.

With regard to preterm infants, the fragile situation in these patients and the small volume of blood pose additional hurdles for reliable testing procedures and need to be addressed in order to gain further insight into the circulating cytokine levels. AlphaLISA based immunoassays offer several advantages over the classical ELISA technology, including reduction of assay time and use of low sample volume, offering an alternative method for the quantification of VEGF and other cytokines in blood samples from infants. Due to the low concentrations of VEGF in blood samples in general and the fact that most VEGF capturing antibodies bind an epitope encoded by exons 1–5, which is present in all isoforms, nothing is known about the distribution of the different isoforms in the blood of preterm infants.

In the present study, we investigated the possibility to use the AlphaLISA system to robustly measure VEGF-A in small blood samples of premature infants. We therefore measured VEGF-A levels in different blood components of healthy adults, as well as of term and preterm infants, both at birth and at the time of acute treatment requiring ROP by using ELISA and AlphaLISA. In order to verify any release of VEGF-A from platelet α-granules, the levels of PF-4 were quantified as well.

## Materials and Methods

The present study followed the tenets of the Declaration of Helsinki and was approved by the Ethics Committee of the Justus-Liebig-University, Giessen (Az 08/13 and 42/15). Informed consent was obtained from the adults, and from the parents of all children participating in the study.

### Subjects

Venous blood samples were taken from ten healthy non-smoker adults, six female and four male, aged 26 to 51 years (demographics of all subjects involved in the study are shown in Table 1). Serum from whole blood (S) and plasma from citrate blood (CB_P) were obtained. Serum from recalcified citrate blood (rS) was also generated as described below.

**Table 1:** Demographic data and blood sample collection.

Group	samples (n)	Age at birth (GA, weeks)	Age at sample collection	Blood sample characteristics
Cohort I: preterm infants	6	25–35	25–35 PMA	4–6 ml CB-Cord Blood
Cohort II: infants with acute ROP	12	23–29	34–44 PMA	<0.1 ml WB, CB
Adult volunteers	10		26–51 years	8–10 ml WB, CB

GA: gestational age in weeks; PMA: postmenstrual age at sample collection in weeks; WB: whole blood; CB: citrate blood.

CB_P of venous cord blood samples from preterm infants with a GA between 25 and 35 weeks (n = 6) were also included in the study. Samples were collected at delivery and CB_P and rS were immediately obtained.

Serum and CB_P samples from 12 infants with acute treatment-requiring ROP taken prior to the intravitreal bevacizumab (IVB) administration were analysed (for details, see Table 1). All infants had developed stage 3+ ROP in zone II and were treated in the following days.

### Blood withdrawal procedures

Whole blood (Table 1) was collected into serum collection tubes (S Monovette, Fa. Sarstedt). The blood was allowed to clot by incubation for at least one hour at room temperature. Tubes were centrifuged at 3000 × g for 15 min. Serum was collected, aliquoted and stored at −80 °C until use. Venous blood was also collected into vacutainers with sodium citrate as anticoagulant (BD Vacutainer, Fa. BD Diagnostics) and CB_P was obtained within the first hour after collection. Briefly, samples were centrifuged at 3000 × g for 15 min at room temperature; plasma was collected, aliquoted and stored at −80 °C until use. In order to determine the influence of platelet activation on the measurements of VEGF-A in CB_P, rS was prepared from one part of the anticoagulated blood samples from healthy adults and cord blood samples from preterm infants. Briefly, 25 mM calcium chloride (Fa. Merck) was added and the blood was incubated at least 90 min at room temperature until a clot was formed. The sample was centrifuged at 3000 × g for 15 min at room temperature, serum from the resulting recalcified blood was collected, aliquoted and stored at −80 °C until use.

### ELISA

VEGF-A and PF-4 concentrations were measured in S, rS and CB_P by Enzyme-linked Immunosorbent Assay (DuoSet ELISA, Fa. R&D Systems) according to the manufacturer’s instructions. This assay employs the quantitative sandwich enzyme immunoassay technique with a specific anti-analyte (VEGF or PF-4) capturing monoclonal antibody and an enzyme-linked anti-analyte detection polyclonal antibody. According to the manufacturer´s information, the VEGF-A capturing antibody recognizes an epitope encoded within exons 3 and 4, which is present in all isoforms. Nunc-Immuno clear standard modules (eight-well strip, MaxiSorp, Fa. Thermo Scientific) were used for the solid phase. Assays were conducted at 18 °C with gentle shaking (500 rpm) until the addition of the substrate solution. After coating and blocking the strips, standards, controls and samples (100 µl/well) were pipetted into the wells in duplicate. For PF-4 quantification, a dilution of S (1:100000), rS (1:50000) and CB_P (1:3000) was required. After an incubation and a wash step, a biotinylated antibody specific against either VEGF or PF-4 was added to each well. Following a thorough wash, the plate was incubated with streptavidin-horseradish-peroxidase and washed again. A substrate solution was added and color developed in proportion to the amount of analyte bound. The color development was subsequently stopped and the optical density determined by using the Infinite M1000 PRO plate reader (Fa. TECAN) at 450 nm with wavelength correction. All samples were used in duplicates, coefficient of determination for standard values was always above R^2^ 0.99. Blank was subtracted from standards, controls and samples. A standard curve was established by plotting the logarithm of the mean absorbance for each standard against the logarithm of the concentration. VEGF-A and PF-4 concentrations were reported as pg/ml and μg/ml, respectively.

### AlphaLISA

In all samples the VEGF-A concentration was measured by AlphaLISA immunoassay (Fa. Perkin Elmer) according to the manufacturer’s instructions. According to the manufacturer, one of the two capturing antibodies (clone 26503) recognizes similarly an epitope encoded within exons 1–5, which is present in all isoforms. Briefly, standards, controls and samples (5 µl/well) and anti-VEGF acceptor beads were pipetted into wells of a half-area 96-well plate in duplicate. After incubation, a biotinylated anti-VEGF antibody was added. Following an incubation step, streptavidin-donor beads were added. Light emission was measured using the Infinite M1000 PRO plate reader (Fa. TECAN). The average count value for the background wells was subtracted from standards, controls and samples. A standard curve was generated by plotting the AlphaLISA counts for each standard versus the concentration. All samples were used in duplicates, coefficient of determination for standard values was always above R^2^ 0.99.

### Statistical analysis

Statistical analyses were performed using SigmaPlot software (Fa. Systat Software). The ANOVA one-way analysis of variance was used to compare growth factor concentrations among the groups. The correlation between S and rS samples was determined by multiple linear regression. For samples containing no detectable levels of VEGF-A, a concentration of 0.1 pg/ml was assigned. P values < 0.05 were considered to be statistically significant.

### Ethical approval

All procedures performed in studies involving human participants were in accordance with the ethical standards of the institutional and/or national research committee and with the 1964 Helsinki declaration and its later amendments or comparable ethical standards. Informed consent was obtained from all individual participants included in the study.

## Results

### Analysis of the concentration of VEGF-A and PF-4 in different compartments of blood samples from healthy adults

In a first step, we analysed the concentrations of VEGF-A in different blood components of healthy adult volunteers. In order to monitor cytokine release after platelet activation, the levels of PF-4 were determined as well. Serum, rS and plasma from blood samples withdrawn with sodium citrate as anticoagulant were tested.

### VEGF-A and PF-4 levels in plasma and serum samples of healthy volunteers

**Figure 1:**
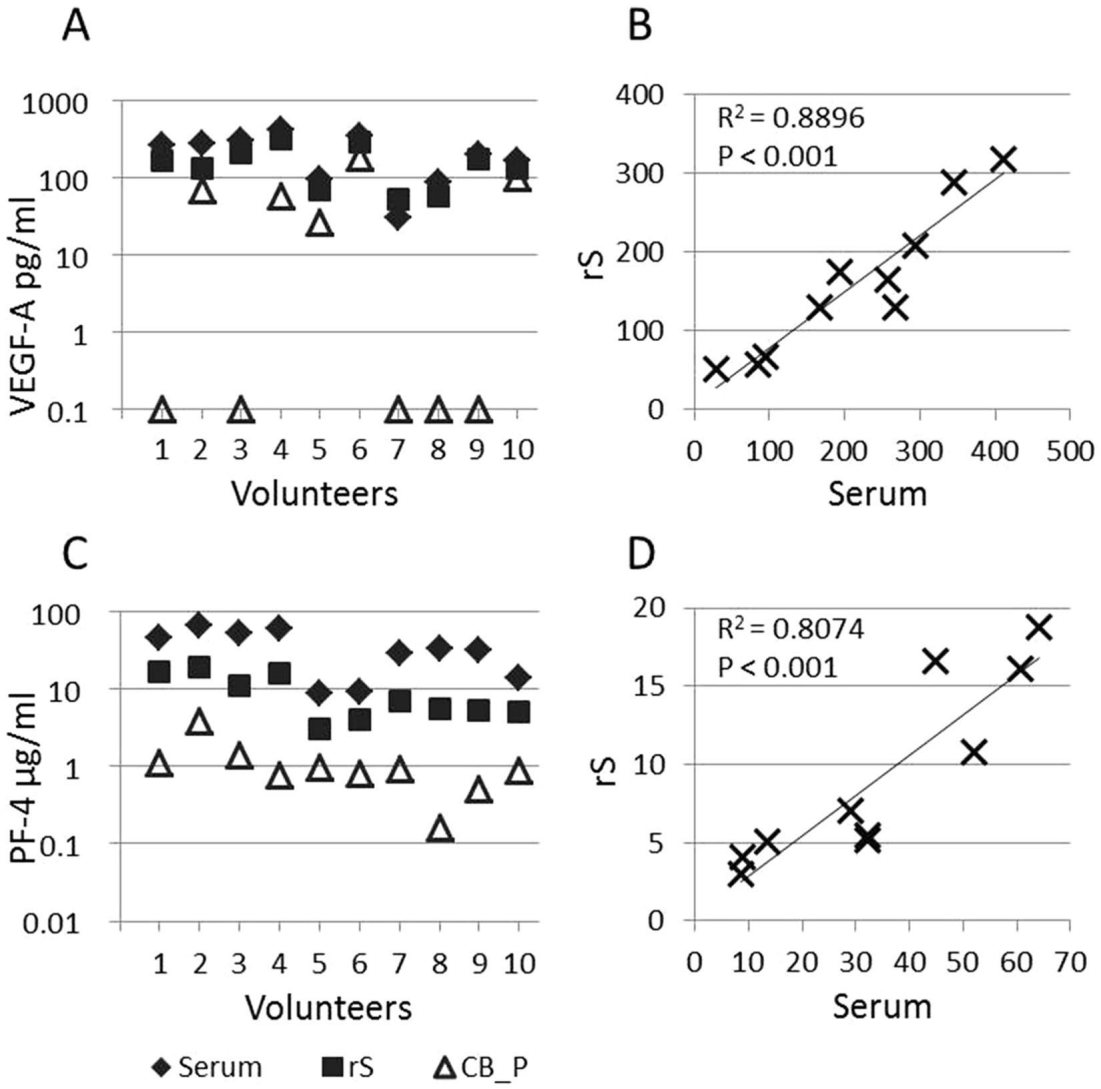
Growth factor levels in blood samples of healthy volunteers. VEGF-A (**A**) and PF-4 (**C**) levels in blood-derived serum (S), serum from recalcified citrated blood (rS) and plasma from citrated blood (CB_P) are indicated. Correlation analyses were carried out for levels of VEGF-A (pg/ml) (**B**) and PF-4 (µg/ml) (**D**) between S and rS. 0.1 pg/ml VEGF-A: VEGF-A concentration below detection level of the assay.

The VEGF-A values in plasma from citrated blood determined by ELISA or AlphaLISA assays ranged from below detection level to 181 pg/ml. An appreciable number of samples (50%) showed no detectable levels of VEGF-A (Fig. 1A). In contrast, the levels in serum and recalcified serum were substantially higher and detectable in all samples, which can be explained by platelet activation.

PF-4 values in citrated plasma samples were between 0.16–3.88 µg/ml. PF-4 concentrations in serum and rS samples were substantially higher (8.61–64.0 and 3.01–18.75 µg/ml, respectively), which is also explained by platelet activation (Fig. 1C).

We verified the release of VEGF-A and PF-4 from platelet α-granules by measuring their concentration in platelet suspensions after either activation or lysis. Platelets were isolated from blood samples, counted and divided in equal parts. In both cases high concentrations of these cytokines were quantified. However, a correlation between platelet counts and concentrations of VEGF-A in serum was not found (data not shown), possibly related to the different amounts of VEGF stored in α-granules in different samples.

### Correlation between levels of VEGF-A and PF-4 in serum from whole blood and serum from recalcified citrated blood

The observation that the concentration of both, VEGF-A and PF4, was increased in serum samples due to platelet activation was further corroborated by the observation that these cytokines showed increased concentrations, similar to real serum levels, in serum made from recalcified blood samples. This correlation was significant for the two cytokines (Fig. 1B,D).

### Correlation between VEGF-A and PF-4 in plasma and serum

We observed a gradual increase of PF-4 concentrations from plasma samples through recalcified serum to serum (Fig. 1C). This cytokine is present at very low levels in citrate plasma, while the levels in serum are much higher, indicating the gradual increase with platelet activation. Since PF-4 levels never surpassed 4 µg/ml in healthy adult plasma samples, and concentrations in serum started at 8 µg/ml, all samples with PF-4 levels below 5 µg/ml can be considered as having no platelet activation beyond what can be tolerated for reliable VEGF-A measurements.

#### Cytokine levels in cord blood of preterm infants

In a second step and with the aim of obtaining information about the concentration of these cytokines in blood samples from preterm infants, we collected cord blood samples from preterm infants at delivery (Table 1, cohort I), obtaining enough volume to isolate CB_P and to generate rS.

VEGF-A and PF-4 values in plasma were in the range previously found in adult volunteers (Table 2). As expected, concentrations of VEGF-A and PF-4 in rS were significantly higher compared to plasma levels. We verified again the release of VEGF-A and PF-4 from platelet α-granules as described before. A correlation between platelet counts and concentrations of VEGF-A in recalcified serum was not found (data not shown).

**Table 2:** Growth factor levels in blood samples from preterm infants.

Sample	Level, Mean (range)	
	VEGF (pg/ml)	PF-4 (µg/ml)
**Cohort I: preterm infants**		
rS	362.26 (155.83–555.78)	5.49 (2.77–9.92)
CB_P	10.08 (n.d. – 36.19)	1.59 (0.53–3.51)
**Cohort II: infants with acute ROP**		
S	447.75 (152.52–1045.27)	19.54 (6.10–38.68)
CB_P	89.04 (19.50–245.91)	1.11 (0.09–3.29)

Abbreviations: S: serum; rS: serum from recalcified citrate blood; CB_P: plasma from citrate blood; n.d.: not detectable.

#### Comparison of ELISA and AlphaLISA for measurement of VEGF concentrations

Both the ELISA and the AlphaLISA for the quantification of VEGF-A are immunoassays with a sandwich based format, high sensitivity (LDL: 9 pg/ml and 2.2 pg/ml respectively) and reproducibility (Intra-Assay and Inter-Assay Precision: CV% < 10% in both cases). In both cases, the capturing antibody recognizes an epitope within the region of exon 1–5, which is present in all known VEGF-A isoforms. Therefore, differentiation between isoforms is not possible with either assay. The low sample volume required in AlphaLISA (5 µl/Well; ELISA sample volume: 100 µl/Well) allows to use very small volume samples, which is especially important in extremely small infants with critical clinical conditions.

**Figure 2:**
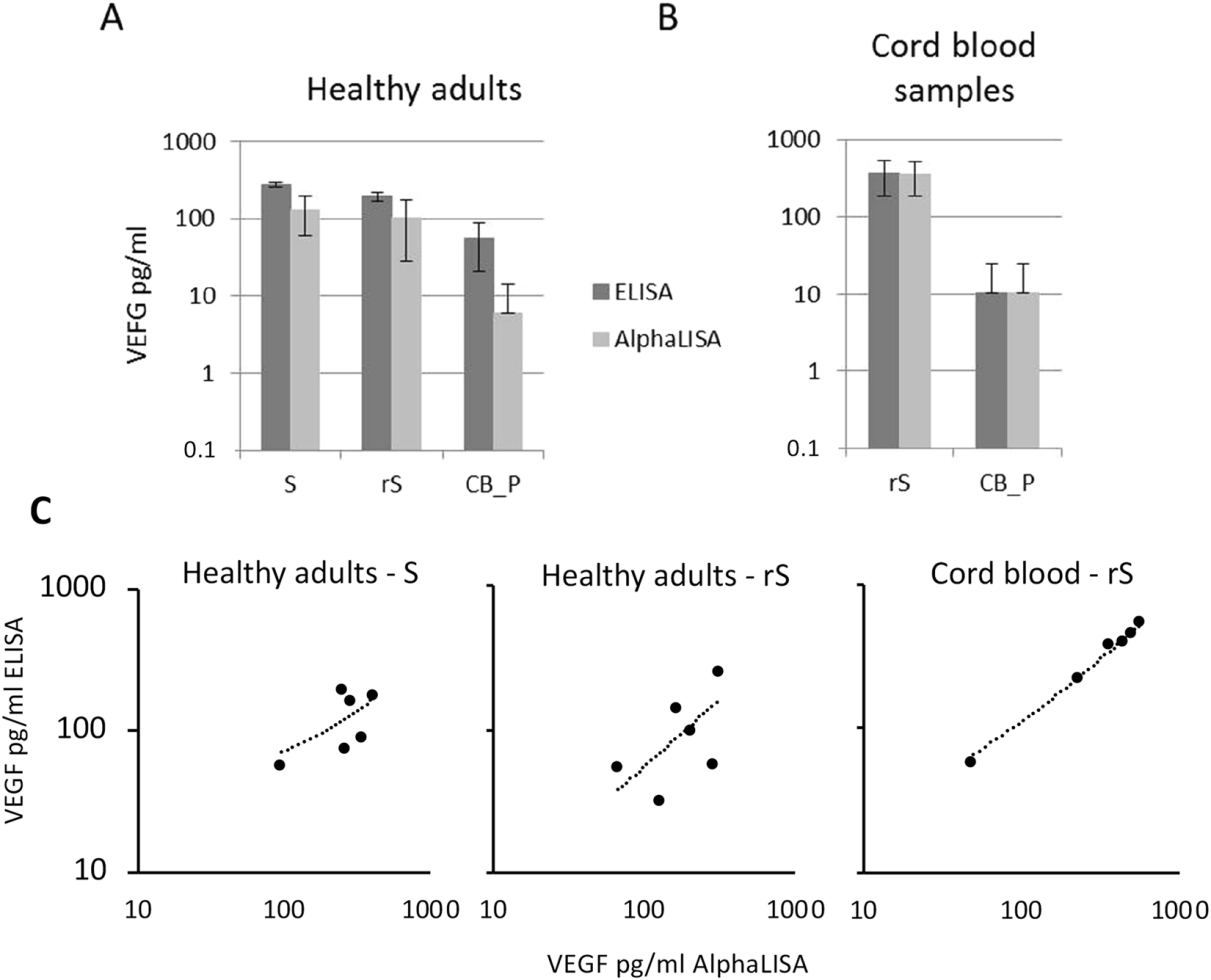
VEGF-A levels in S, rS and CB_P. Samples from healthy volunteers (**A**) and in rS and CB_P from cord blood samples (**B**) were quantified using ELISA and AlphaLISA and mean values are presented. (**C**) Scatter plot visualization of VEGF A levels obtained from ELISA and AlphaLISA assays in rS and S samples from adults and from cord blood samples.

In order to verify the performance of these two assays we compared the measured VEGF-A concentration in the samples of different blood compartments from healthy adults and in the CB_P and rS samples from preterm infants (cohort I). The AlphaLISA assay detected lower levels of VEGF-A compared to ELISA in the samples from healthy adults but the differences in the mean values among the groups were not high enough to find a statistically significant difference (Fig. 2A). In the case of CB_P and rS samples from preterm infants, similar amounts of VEGF-A were quantified with both assays (Fig. 2B). Pairwise scatter plot analysis in S and rS samples of adult and cord blood samples revealed comparable data (Fig. 2C). Since some of the plasma values obtained from adult and cord blood sample were below the level of detection, scatter plot visualization was not possible.

#### VEGF-A and PF-4 levels in blood samples from infants with acute treatment-requiring retinopathy of prematurity (ROP)

Serum and plasma samples were obtained from 12 patients with acute treatment-requiring ROP prior to the IVB (Table I, cohort II). At the time of sample collection, all children had developed a stage 3+ ROP in posterior zone II and were treated the following day. Samples were assayed using AlphaLISA to determine the concentration of VEGF-A. As a reference of platelet activation, the levels of PF-4 were quantified in parallel. In all samples, VEGF-A could be measured with values ranging from 152.52 to 1045.27 pg/ml in serum and 19.50 to 245.91 pg/ml in plasma (Table 2, cohort II). PF-4 concentrations in plasma and serum ranged between 0.09 and 3.29 µg/ml and between 6.10 and 38.68 µg/ml, respectively (Table 2, cohort II), indicating that the VEGF-A plasma levels represented free VEGF-A due to absent platelet activation.

## Discussion

In this study, we show that the AlphaLISA assay is well suited for the quantification of VEGF-A in small sample volumes (just 10 µl of plasma or serum), enabling clinician scientists to obtain valuable data from blood samples of premature infants, where iatrogenic anaemia is an issue.

VEGF-A can be measured in plasma or serum samples. In plasma, VEGF-A levels represent the free circulating form of the cytokine, whereas in serum, platelet-derived VEGF-A contributes to the overall cytokine concentration. In order to obtain reliable measurements of VEGF-A in plasma, platelet activation needs to be avoided. In our study, when VEGF-A and PF-4 levels were compared in different samples (e.g. plasma vs. serum), it was obvious that platelet release reactions significantly contributed to alterations/increase in the level of these cytokines (Fig. 1). We found a significant correlation between the values of VEGF-A and PF-4 in serum from whole blood and serum from recalcified citrated blood, suggesting that recalcified serum could serve as an alternative to estimate the concentration of VEGF-A in serum and to monitor its release from platelets after *in vitro* activation in cases where just a citrate blood sample is available.

In this study, the concentration of VEGF-A in CB_P samples from healthy volunteers and in cord blood from preterm infants was often below detection level. In other reports, similar results were observed^22–24^ suggesting that the level of free circulating VEGF-A is often, but not always, very low in healthy subjects. In plasma samples from infants with acute treatment-requiring ROP, the concentration of VEGF-A was always above detection level, indicating that the levels of VEGF in the circulation may well be correlated to those in the eye. Further studies analysing this issue are ongoing. Of note, the levels of VEGF-A in preterm infants can be confounded by other circulating molecules binding to VEGF, such as sFlt, a soluble form of the VEGF receptor 1, that is known to be increased in preeclampsia^25^. This issue is a general limitation of measuring VEGF levels in the circulation, which cannot be addressed by the assays presented in this study.

When we compared the measured levels of VEGF-A in cord blood samples from preterm infants by using either ELISA or AlphaLISA, we did not find a significant difference. Based on this result and because our aim was to reduce phlebotomy losses by collecting small amounts of blood from the fragile infants, we determined the VEGF-A levels in plasma and serum from infants with treatment required ROP using AlphaLISA. VEGF-A concentrations ranged from 19.50 to 245.91 pg/ml in plasma and from 152.52 to 1045.27 pg/ml in serum. Similar results have been reported in other studies, albeit with some variation, in which the levels of VEGF were quantified by ELISA (Table 3)^10,13,14,26–28^. These data support the validity of using AlphaLISA as an alternative method to ELISA for the quantification of VEGF-A. The reason for the particularly good correlation between the two assays in cord blood samples in contrast to adult blood samples remains elusive, it may well be that adult blood samples contain higher amounts of confounding factors influencing the results with either assay.

**Table 3:** VEGF concentration in blood samples from preterm infants with acute ROP before treatment.

Author (s), year	VEGF levels (pg/ml)		Patients (n)
	Plasma	Serum	
Zhou *et al*., 2016^a^	46.04 ± 9.40^*^		11
Hong *et al*., 2015^a^	2050 ± 3000^*^		6
Wu *et al*., 2017^a^		440.6 (156.5–807.5) 351.8 (281.9–634.4)	64
Wu *et al*., 2015^a^		226 (190–568)	8
Sato *et al*., 2012^a^		1628 ± 929	5
Stahl *et al*., 2018^a^	26,32 (7.8–59.58)		16
This study^b^	89.04 (19.50–245.91)^**^	447.75 (152.52–1045.27)	12

VEGF concentrations were measured by using ELISA^a^or AlphaLISA^b^; *EDTA plasma; **Citrate plasma.

In several studies, the levels of VEGF-A in plasma or in serum have been measured in samples from preterm infants following three aims: (1) to analyse the association between VEGF-A concentrations and physiological retinal maturation, (2) the association between VEGF-A concentrations and the development of ROP (treatment requiring or spontaneously regressing), and (3) to control the systemic concentration of VEGF-A before and after intraocular anti-VEGF therapy^8–12^. In particular, escape of bevacizumab or other anti-VEGF molecules from the eye into the systemic circulation causes reduction of the systemic VEGF-A levels even below detection level. The impact of this inhibition on the physiological development of infants is unclear so far and it is currently being discussed^8,29,30^. Therefore, the monitoring of systemic VEGF-A levels, especially in infants treated with anti-VEGF-A agents, should be considered. The potential interaction of anti-VEGF molecules present in the blood samples after intraocular anti-VEGF treatment with the VEGF binding factors in the ELISA and AlphaLISA assays may represent a significant limiting factor and is currently under investigation.

In conclusion, our study demonstrates that the AlphaLISA assay represents an appropriate immunoassay platform for the quantification of VEGF-A. The tiny amount of blood needed (20–50 µl whole blood) can be acquired at the time of routine blood drawings and allows to screen VEGF-A levels even in very preterm infants without putting an additional risk on the already fragile organism. However, further studies are required to validate the role of systemic VEGF-A as a clinically relevant biomarker for the management of acute ROP.
